# Restoring Skeletal Muscle Health through Exercise in Breast Cancer Patients and after Receiving Chemotherapy

**DOI:** 10.3390/ijms25147533

**Published:** 2024-07-09

**Authors:** Inês Aires, José Alberto Duarte, Rui Vitorino, Daniel Moreira-Gonçalves, Paula Oliveira, Rita Ferreira

**Affiliations:** 1 LAQV-REQUIMTE, Department of Chemistry, University of Aveiro, 3810-193 Aveiro, Portugal; iaa@ua.pt (I.A.); ritaferreira@ua.pt (R.F.); 2 CITAB, Inov4Agro, University of Trás-os-Montes and Alto Douro (UTAD), Quinta de Prados, 5000-801 Vila Real, Portugal; pamo@utad.pt; 3 CIAFEL, and Laboratory for Integrative and Translational Research in Population Health (ITR), Faculty of Sports, University of Porto (FADEUP), 4200-450 Porto, Portugal; jose.duarte@iucs.cespu.pt (J.A.D.); danielmgon@fade.up.pt (D.M.-G.); 4 UCIBIO-Applied Molecular Biosciences Unit, Translational Toxicology Research Laboratory, University Institute of Health Sciences (1H-TOXRUN, IUCS-CESPU), 4585-116 Gandra, Portugal; 5 iBiMED, Department of Medical Sciences, University of Aveiro, 3810-193 Aveiro, Portugal

**Keywords:** mammary tumorigenesis, physical activity, aerobic exercise, resistance training, muscle wasting, metabolic remodeling

## Abstract

Breast cancer (BC) stands out as the most commonly type of cancer diagnosed in women worldwide, and chemotherapy, a key component of treatment, exacerbates cancer-induced skeletal muscle wasting, contributing to adverse health outcomes. Notably, the impact of chemotherapy on skeletal muscle seems to surpass that of the cancer itself, with inflammation identified as a common trigger for muscle wasting in both contexts. In skeletal muscle, pro-inflammatory cytokines modulate pathways crucial for the delicate balance between protein synthesis and breakdown, as well as satellite cell activation and myonuclear accretion. Physical exercise consistently emerges as a crucial therapeutic strategy to counteract cancer and chemotherapy-induced muscle wasting, ultimately enhancing patients’ quality of life. However, a “one size fits all” approach does not apply to the prescription of exercise for BC patients, with factors such as age, menopause and comorbidities influencing the response to exercise. Hence, tailored exercise regimens, considering factors such as duration, frequency, intensity, and type, are essential to maximize efficacy in mitigating muscle wasting and improving disease outcomes. Despite the well-established anti-inflammatory role of aerobic exercise, resistance exercise proves equally or more beneficial in terms of mass and strength gain, as well as enhancing quality of life. This review comprehensively explores the molecular pathways affected by distinct exercise regimens in the skeletal muscle of cancer patients during chemotherapy, providing critical insights for precise exercise implementation to prevent skeletal muscle wasting.

## 1. Introduction

Breast cancer (BC) is the most commonly diagnosed type of cancer in women worldwide [1]. With increased life expectancy, improved diagnostic techniques and cancer registration, BC incidence has been on the rise globally. BC represents a vast and heterogeneous group of tumors, each with distinct cellular and molecular features, leading to varying clinical presentations and responses to therapy [2]. Tailoring treatment approaches to the specific subtype of BCa is crucial, as each subtype exhibits a unique response to therapy. While targeted therapies have emerged, cytotoxic drugs remain the most effective means of treating BC metastasis [3]. BC patients typically receive chemotherapy regimens that include antineoplastic drugs, such as the combination chemotherapy regimen with cyclophosphamide, methotrexate, and 5-fluorouracil, anthracyclines (doxorubicin, epirubicin), and taxanes (paclitaxel, docetaxel). These regimens can be administrated alone or concurrently with radiotherapy, surgery, and/or hormonal therapy [4]. However, these antineoplastic agents also affect normal cells, leading to chemotherapy’s well-known side effects such as secondary leukemia and cardiomyopathy, which can result in congestive heart failure [5]. Skeletal muscle wasting, a side effect of cancer and chemotherapy, is frequently underestimated, despite its significant impact on the quality of life of patients, wherein fatigue is recognized to reduce patient tolerance to cancer therapies [6].

**Figure 1:**
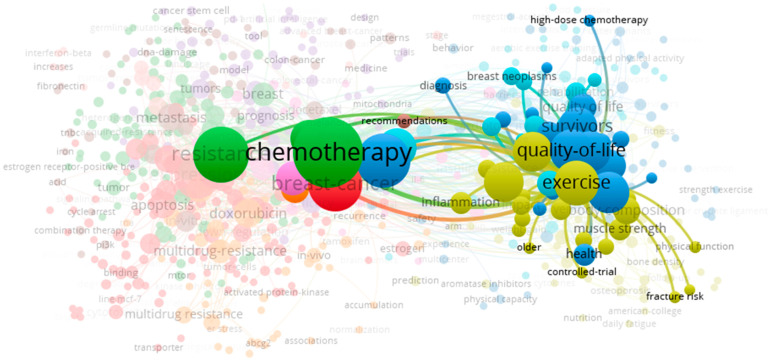
Bibliometric network analysis of common terms implicated in cancer, chemotherapy, and exercise. Node size is associated with the frequency of the term and the edges signalize co-occurring terms. Different colored lines represent clusters of associated terms. The co-occurrence network visualization map was created using VOSviewer (version 1.6.20).

In the pursuit of more effective and less debilitating treatments to complement the multimodal management of BC patients, non-pharmacological therapeutic interventions have been explored, such as diet supplementation [7,8] or physical exercise [9]. In fact, the hypothesis that physical exercise can offer protection against BC dates back to the 1980s [10]. Several potential molecular mechanisms linking physical activity and BC have been proposed, including alterations in the serum levels of sex hormones, insulin, and adipokines, along with changes in molecular pathways associated with inflammation and oxidative stress within skeletal muscle [11]. Nonetheless, the molecular pathways underlying skeletal muscle wasting remain unclear, particularly in the realm of BC chemotherapy. To gain insights into the role of exercise in the management of BC, the VOSviewer tool was used to visualize and analyze bibliometric data on the co-occurring terms “cancer”, “chemotherapy”, and “exercise”. The results presented in Figure 1 highlight that exercise primarily serves to improve patients’ quality of life. Interestingly, certain aspects have received limited attention in research, e.g., cancer- and chemotherapy-related comorbidities such as skeletal muscle wasting. Several factors are also involved in this intricate interplay, which, despite their importance, they often only occupy a peripheral position in the network. These factors encompass exercise intensity, duration, and type, the specific chemotherapy regimen, and individual patient characteristics.

Therefore, a comprehensive understanding of the role that these nuanced factors play in the broader network of exercise in BC could significantly advance the concept of “Exercise is Medicine”. This review aims to overview how different exercise programs may counteract the metabolic adaptations underlying BC and/or chemotherapy-induced muscle wasting and holds the prospect of defining guidelines for prescribing tailored exercise programs in the cancer setting.

## 2. Tailoring Exercise Programs to Address Breast Cancer-Related Body Wasting

Research on the complex interplay between cancer and chemotherapy-induced muscle wasting has supported the beneficial effects of diverse exercise programs. However, as the concept of “Exercise is Medicine” gains increasing recognition, it becomes crucial to understand how to maximize its health benefits. Each patient has specific needs, being challenging to implement “one-size fits all” training program. However, it is extremely difficult to prescribe and implement an exercise training program tailored to the specific needs of each individual. As a result, exercise programs that aim to address cancer-related outcomes, such as fatigue, anxiety, and quality of life (QoL), have shown beneficial effects in clinical trials and are usually employed. Nevertheless, distinct exercise programs have yielded comparable health benefits. For instance, a moderated aerobic training routine performed at least three times *per* week for a minimum of 30 min [12], or a resistance training program conducted at least twice a week with a minimum of two sets of 8–15 repetitions [13], have shown comparable outcomes. Furthermore, combined exercise training, twice a week for 80–90 min, has demonstrated similar effectiveness [12]. Still, not all patients benefit equally from the same types of exercise regimens. Addressing this challenge requires a multifaceted approach when prescribing an exercise program that takes into account a spectrum of factors related to the oncologic condition such as disease stage, as well as factors beyond the oncologic condition. These factors, including age, sleep schedule, nutrition, environment, and physical and mental condition, must all be thoughtfully considered in exercise prescription [14]. 

When establishing an exercise routine, the extent of skeletal muscle wasting must be carefully evaluated. Although both cancer- and chemotherapy-induced muscle wasting may lead to significant weight loss, this change in weight does not necessarily align with alterations in body composition [15]. Patients undergoing chemotherapy may be more prone to severe muscle wasting compared to those experiencing muscle loss solely due to cancer. Patient’s age plays a crucial role in shaping personalized exercise programs, especially in older individuals facing sarcopenia [16,17,18,19]. Additionally, it is worth recognizing that BC patients, including younger ones, may undergo menopause. Those may often experience a more pronounce muscle wasting and may not respond to exercise as effectively as premenopausal ones [20]. Patients experiencing severe muscle wasting or in advanced cancer stages might not be suitable candidates for exercise programs that require a performance level capable of discerning measurable benefits [21]. In fact, determining the optimal timing for prescribing exercise to counteract muscle wasting may be a challenge. While physical activity undeniably offers significant health benefits and is essential across various contexts, its implementation in cancer patients presents unique challenges, due to differences in disease stages that may affect patients’ performance. Ideally, identifying patients at higher risk of developing muscle wasting and intervening at the earliest possible stage would be the ultimate goal. However, predicting and identifying these deleterious muscle wasting conditions may prove challenging [22]. Nevertheless, when feasible, the early initiation of exercise before muscle wasting becomes more pronounced can serve multiple purposes. During these initial phases, exercise may primarily address cancer-induced comorbidities or mitigate the toxic effects associated with the early stages of chemotherapy-induced muscle wasting [5]. In more advanced stages, if feasible, lower impact exercises should be prioritized to prevent exceeded fatigue, potentially compromising overall health and treatment effectiveness during therapy [23].

### Exercise Counteracts Breast Cancer- and Chemotherapy-Induced Body Wasting

Although not as prevalent in BC as in other cancer types like pancreatic, gastric, and lung cancer, body weight loss due, at least in part, to muscle wasting can occur in advanced-stage BC patients [24]. This skeletal muscle loss is a central component of cachexia, a syndrome that significantly contributes to poor performance status and increased mortality among cancer patients [25]. Furthermore, chemotherapy can exacerbate the skeletal muscle atrophy, which is linked to a poor prognostic, and increased mortality and morbidity [25]. Importantly, exercise interventions have demonstrated promise in alleviating muscle atrophy and enhancing the overall well-being of BC patients [26]. However, to maximize the benefits of exercise in the BC setting, it is crucial to gain a better comprehension of its role on the interplay between cancer- and chemotherapy-induced muscle wasting. Such understanding can be achieved through, for example, a comprehensive comparison of the whole-body and skeletal muscle remodeling promoted by various exercise protocols. In fact, it becomes evident that even when engaging in the same type of exercise, minor variations in parameters such as duration or intensity can yield distinct outcomes [27,28,29,30]. Therefore, acknowledging the molecular mechanisms that govern the interplay between distinct exercise programs and BC-induced muscle wasting will pave the way for the development of exercise regimens tailored for BC patients. However, there is a scarcity of studies examining whole-body adaptations in BC patients undergoing chemotherapy and participating in exercise programs, with, to our knowledge, only six studies dedicated to the evaluation of skeletal muscle remodeling. When such investigations do take place, they typically rely on imaging-based approaches, e.g., L3 muscle index based on computerized tomography and functional tests (e.g., assessment of muscle strength and fatigue), with few studies including muscle biopsies (Table 1). In fact, muscle biopsies were conducted in merely two studies [29,30], and molecular data were only reported in one [29]. The absence of muscle biopsy data limits our understanding of the molecular mechanisms of skeletal muscle adaptation to exercise in BC, thereby limiting the prescription of exercise in therapeutic plans due to the lack of robust molecular evidence.

Exercise, regardless of the regimen, has been reported to lower fatigue, depression, and anxiety, improving the overall self-esteem and quality of life of BC patients [41]. When assessing the impact on quality of life, all types of exercise have shown improvements (Table 1). Hence, some studies have included control groups that were also encouraged to follow an exercise routine and were provided with an exercise protocol, albeit without supervision after the post-intervention assessments [27,30]. The majority of the exercise protocols were performed under supervised conditions, which is acknowledged as a key factor for the effectiveness of the exercise regimens [38]. Nonetheless, including exercise in a therapeutic plan for BC patients presents significant challenges. The lack of detailed molecular knowledge about exercise effects in cancer context frequently raises doubts about its potential effectiveness among clinicians and patients. Furthermore, BC patients frequently lack motivation due to increased fatigue. In those cases, group classes can be more beneficial, as they are more enjoyable [42,43] and have shown high attendance rates among women [42,44]. However, the primary limitation may be severe muscle wasting and other cancer comorbidities, which may coincide with more advanced disease stages and aggressive treatments, potentially hindering patients’ capacity to engage in exercise programs [45].

In the BC setting, while reduced food intake can contribute to muscle loss, the hypermetabolic state that increases resting energy expenditure (REE) in patients results in involuntary body weight loss, a hallmark of cachexia [46,47]. BC patients commonly exhibit lower lean body mass levels, as the shifting in metabolism, marked by an increase of catabolic processes while anabolic ones are downregulated, is primarily associated with tumor metabolism [48,49]. In fact, despite the pivotal role of the imbalance between protein synthesis and proteolysis in driving muscle wasting, the well-known “Warburg effect” may also contribute to the increase of REE, particularly in more advanced stages of cancer [49,50]. Additionally, the early menopause experienced by younger BC patients under treatment may not only affect the quality of life but also the body composition. Post-menopause women, characterized by low estrogen levels and an often decline in overall physical activity, may undergo an increase in body fat, particularly visceral adipose tissue [51]. Premenopausal women with BC following chemotherapy also experience a decrease in lean body mass, akin to what is observed in postmenopausal women [16,52]. Exercise, particularly resistance training protocols, have demonstrated significant gains in lean body mass [27,28], making them advisable for BC. Interestingly, some aerobic programs have also shown some lean body mass gains [27,28]. An increase in lean body mass can counteract muscle wasting, yet it might not necessarily result in a rise in REE. In cancer patients, the overall muscle mass will still be lower compared to a healthy individual. In those cases, and considering the overall health condition, the focus is primarily preserving the muscle mass rather than drastically increasing it [53]. Lean body mass can also have an impact on chemotherapy toxicity [54]. Chemotherapy dosages are typically determined based on body surface area (m^2^), considering solely the height and weight. However, if two patients, for example, share identical measurements, the one with higher lean body mass will likely experience fewer chemotherapy-related toxicities. Hence, an exercise protocol that enhances lean body mass levels becomes a valuable therapeutic strategy in this context [27,28]. Exercise, particularly resistance training protocols have demonstrated significant gains in lean body mass [27,28,37]. Interestingly, some aerobic programs have also shown some gains [27,28].

## 3. Exercise Training Counteracts Cancer- and/or Chemotherapy-Induced Muscle Wasting: The Molecular Targets

There are few studies addressing the molecular alterations underlying cancer- and chemotherapy-induced skeletal muscle wasting in humans [29,30], which may be due, in part, to ethical constraints on taking muscle biopsies from BC patients. Indeed, the diagnosis of muscle wasting in BC patients is often underestimated, and even when it is recognized, it usually occurs at advanced stages of the disease, which poses a greater challenge for its effective clinical management. Moreover, determining the extent of muscle atrophy in cancer patients is a difficult task, as the decrease in total body weight is not always accompanied by the loss of skeletal muscle mass [15]. Animal models offer the advantage of allowing a comprehensive examination of the remodeling of whole muscle tissue in response to cancer and/or therapy. This analysis can be performed without potential biases from factors such as diet or unrelated therapies [26,55]. However, to our knowledge, there are no studies that have investigated the impact of exercise on the interplay between BC and chemotherapy in animal models of mammary tumorigenesis. Nevertheless, it is possible to gain insights into the therapeutic effect of exercise on the molecular mechanisms underlying cancer- and chemotherapy-induced muscle wasting by focusing on research developed exclusively in the context of BC or chemotherapy. Translation of the molecular findings underlying muscle wasting from other cancer models requires more cautious interpretation, as evidence of a direct link to BC remains hypothetical until further dedicated research is conducted.

### 3.1. Exercise-Induced Remodeling of Muscle Fiber Phenotype in Cancer-Induced Muscle Wasting

Research in animal models of BC has shown muscle atrophy in both muscle fibers type I and type II [56]. However, the glycolytic fast-twitch type II fibers seem to be more prone to wasting [57], characterized by an accentuated decrease of fiber size [26]. Following carboplatin treatment, BC animals exhibited a decrease of all muscles mass, although only *tibialis anterior* showed a reduction in fiber cross-sectional area (CSA) [58]. Similar outcomes were observed in rats treated with doxorubicin, although healthy animals were enrolled and not those with BC or other types of cancer [59]. The results remained consistent across other types of cancer, suggesting that the decrease in fiber CSA, and consequently muscle atrophy, may not be linked to cancer type, but rather to tumor growth and chemotherapy administration [60]. This association may raise questions about the underlying mechanisms, which have been proposed to involve inflammation, increased skeletal muscle oxidative stress, mitochondrial dysfunction, and apoptosis [26,61,62].

Distinct muscles respond differently to various types of exercise in wasting conditions. In doxorubicin-treated healthy animals, no differences in *soleus* mass were observed following aerobic exercise (progressive treadmill training for 2 weeks) [36] or resistance (jump to reach food and water with a progressively increasing height (until 8 inches) for 2 weeks, representing ~70% VO_2max)_ [35]. However, for the *extensor digitorum longus* (EDL) muscle the same resistance exercise program was shown to promote mass gain [35]. In aerobic exercise protocols, the outcomes reported in the literature depend on the protocol’s duration. While a progressive treadmill protocol performed for 2 weeks yielded the same results as the resistance exercise protocol, animals in a 10-week protocol (progressive treadmill starting at 20 m/min, 20 min/day and increasing until 30 m/min, 60 min/day, 18° incline, representing ~75% VO_2max_) showed a decrease in EDL mass [35,36]. Notably, and although the resistance exercise-induced hypertrophy is more prominent in glycolytic type II fibers, an increase in fiber CSA has been reported in all fiber types, resulting in an overall enhancement of muscle force [63,64]. In contrast, aerobic exercise promotes a shift towards increased recruitment of oxidative type I fibers [61]. This fiber switch is linked to metabolic alterations towards an oxidative phenotype [65,66,67], being better positioned to alleviate muscle fatigue rather than increase overall muscle force [35].

### 3.2. Exercise Counteracts Oxidative Stress and Inflammation on Cancer and Chemotherapy-Induced Muscle Wasting

**Figure 2:**
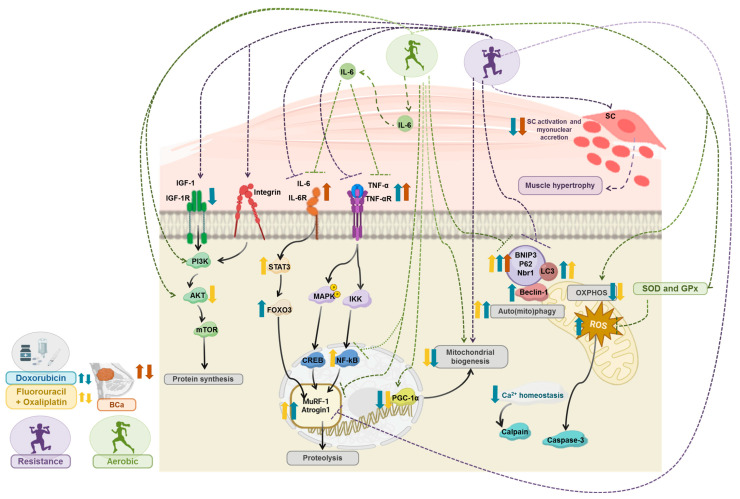
Overview of the key molecular mechanisms involved in Breast Cancer (BC) and chemotherapy-induced muscle wasting, and the impact of different exercise types on them. The BC effect is represented in brown, chemotherapy in blue, aerobic exercise in green, and resistance exercise in purple. Figure was created with BioRender.com.

Chemotherapy-induced oxidative stress has been extensively hypothesized as a key molecular mechanism underlying skeletal muscle wasting in the context of cancer treatment. This increase in oxidative stress seems to be linked to a decrease in the content of antioxidant systems triggered by doxorubicin, a phenomenon also observed in cardiac cells [68]. Furthermore, given the high metabolic activity of skeletal muscle, there is an increased generation of reactive oxygen species (ROS), primarily associated with mitochondrial dysfunction, which is marked by decreased mitochondrial respiratory chain activity and ATP synthesis [6,59,69,70]. Doxorubicin-related production of ROS occurs mainly at electron transport chain (ETC) complex I, where this drug undergoes a single-electron reduction reaction. The high affinity of doxorubicin for the inner mitochondrial membrane, particularly for the phospholipid cardiolipin, facilitates its binding and subsequent reduction. This reaction results in the removal of electrons from the ETC, disrupting oxidative phosphorylation and ATP production [71,72]. The consequent decline in the ATP generation will ultimately lead to skeletal muscle’s metabolism disruption, resulting in muscle weakness [73]. Moreover, the increased ROS levels induced by doxorubicin and other antineoplastic drugs may trigger lipid peroxidation, which can disturb the integrity and function of the cell membrane, with impact on muscle contractile function [74]. ROS may also impair the contractile process by interfering with intracellular and mitochondrial Ca^2+^ homeostasis, leading to impaired muscle force generation and contractility [75,76]. Moreover, the elevation in ROS levels can modulate signaling pathways, such as NF-κB signaling pathways, which promote muscle protein breakdown and inhibit muscle protein synthesis [76]. In fact, several proteolytic systems, such as calpains, caspases, the ubiquitin-proteasome system (UPS), and autophagy, have been reported to be activated in skeletal muscle in response to chemotherapy, stimulating the STAT3 pathway and Akt-mediated phosphorylation of FOXO3, leading to the overexpression of ubiquitin E3 ligases on *soleus* and *gastrocnemius* muscles [32,77,78] (Figure 2).

The role of exercise in oxidative stress has caught significant attention because exercised-induced increase in ROS may activate signaling pathways and promote skeletal muscle adaptation to exercise [79]. ROS signaling plays a role in modulating specific pathways, such as activating NF-kB and MAPK. It has been proposed that ROS may trigger adaptive responses through these redox-sensitive signaling pathways to maintain cellular oxidant-antioxidant homeostasis during exercise. In short time periods, ROS activates signaling pathways that modulate cellular adaptations, protecting against future stresses. In longer time periods, ROS signaling may result in the chronic activation of pathways that promote proteolysis, potentially leading to cell death [80]. However, the exercise-induced skeletal muscle adaptation mediated by ROS extends beyond these pathways, as it has been proposed that ROS production is necessary for peroxisome proliferator-activated receptor-γ coactivator-1α (PGC-1α) expression, a crucial player in aerobic exercise adaptation [81]. Hence, given the pivotal role of ROS signaling, one could anticipate that engaging in exercise training may lead to an escalation in ROS levels in the skeletal muscle of BC patients, particularly when baseline ROS levels are already elevated. However, endurance exercise (progressive treadmill 5 days/week, increasing 10 min/day until 60 min/day at 30 m/min, for 2 weeks, representing ~70% VO_2max_) was reported to increase the expression of antioxidant enzymes such as superoxide dismutase, and glutathione peroxidase in the *soleus* of doxorubicin-treated rats while simultaneously decreasing the activity of calpains and caspase-3 [34], potentially counteracting the muscle atrophy induced by this anticancer drug [26].

On the other side, auto(mito)phagy is also activated in inflammatory conditions and associated with metabolism disruption and skeletal muscle mass loss [82,83,84]. This highly selective and regulated process involves specific signals such as p62, Bnip3, Nbr1, which are composed of a cargo-binding domain that recognizes and attaches to organelles, and a LC3-interacting region (LIR) responsible for recruiting and binding to autophagosome membrane proteins [85]. Increased auto(mito)phagy leads to muscle atrophy, while a decrease in this process may result in muscle weakness and myofibers degeneration [86,87]. Notably, in animals treated with doxorubicin, there was a decrease in the expression of beclin-1 during endurance exercise (progressive treadmill 5 days/week, increasing 10 min/day until 60 min/day at 30 m/min, for 2 weeks, representing ~70% VO_2max_) compared to sedentary ones [34]. In doxorubicin-treated rats submitted to another aerobic protocol (treadmill 5 days/week, 40–60 min/day, at 60% maximum speed), the autophagy genes were analyzed concurrently with STAT3, which was regulated by the pro-inflammatory IL-6, and decreased levels were found following exercise [32,77]. Additionally, in mice inoculated with Colon26 carcinoma cells, other endurance protocol (motorize wheel, 5 days/week, 5 m/min for 15 min, increasing 11 m/min for 45 min) was shown to decrease Bnip3 levels. Concurrently, the decrease in autophagy was accompanied by an increase in mitochondrial function, as evidenced by the upregulation in *Mfn2* gene expression, indicating fusion, and no changes in *Fis1* and *Mfn1* gene expression, indicating fission [33]. As for resistance training (ladder-climbing protocol 3 days/week, load at 75% body-weight that increased according to performance), in mice inoculated with breast carcinoma cells, a decrease in autophagy proteins was observed, especially LC3B-II and beclin-1 [88] (Figure 2). Under normal conditions, the auto(mito)phagy process helped maintaining a pool of healthy mitochondria by eliminating the defective ones [84]. Hence, in cancer settings, the observed exercise-induced decrease of auto(mito)phagy may reflect healthier mitochondria that do not need to be eliminated to avoid the toxicity of their accumulation [33].

Exercise training can promote an increase in circulating skeletal muscle-derived IL-6, known for its anti-inflammatory activity, as it inhibits the action of pro-inflammatory cytokines like TNF-α and promotes the synthesis of anti-inflammatory cytokines, particularly IL-10 [89]. The peak of anti-inflammatory IL-6 typically occurs at the end or shortly after exercise, followed by a rapid decline in its levels [90]. This peak is more pronounced in endurance exercise with longer duration, particularly when larger muscle groups are engaged. Interestingly, different effects are anticipated for distinct exercise intensities, with high-intensity exercise more effective at counteracting the effects of pro-inflammatory cytokines compared to low-intensity exercise [91]. Resistance exercise for 16 weeks (3 times/week,15 exe. 3 sets, 20 reps, increased weight if 20 reps are performed) was reported to decrease the circulating levels of inflammatory cytokines such as IL-6, TNF-α, IL-8, and IL-1, even in postmenopausal BC patients [92]. In fact, resistance exercise promoted a decrease in the expression of Toll-like receptor-4 (TLR-4) in monocytes/macrophages [93], diminishing the TLR-4-induced pro-inflammatory cytokines [94]. Thus, by reducing TLR-4 expression in monocytes/macrophages, resistance exercise may counteract cancer- and chemotherapy-induced muscle wasting. Aerobic exercise did not induce significant changes in TLR-4 expression in the *gastrocnemius* of doxorubicin-treated healthy animals [32], possibly reflecting no alterations in macrophage infiltration in skeletal muscle following treatment. On the other hand, combined exercise has demonstrated only a decrease in IL-6 levels in mice inoculated with Colon26 carcinoma cells or Lewis Lung carcinoma cells [67].

### 3.3. Metabolic Remodeling and Hormonal Influence on Skeletal Muscle Response to Exercise in Cancer Settings

Dysregulation of glucose metabolism has been observed in BC patients [95] and associated with the pro-inflammatory status that plays a critical role in the development of insulin resistance [96]. Exercise, particularly aerobic exercise due to its anti-inflammatory effect, may improve insulin sensitivity in skeletal muscle, with an impact on its metabolism. In fact, aerobic exercise has received more attention in the literature compared to resistance training in the management of muscle wasting associated to chronic diseases such as cancer. Endurance training has shown favorable effects on muscle insulin sensitivity through the modulation of GLUT4 and glycogen synthase expression, as well as glucose oxidation [97]. Regarding resistance exercise, no studies using animal models of cancer were found evaluating insulin sensitivity. However, resistance exercise seems to modulate insulin sensitivity in cancer patients by preserving or enhancing lean body mass [98,99]. While these results were initially demonstrated in diabetic patients, similar results were reported in BC patients [43]. However, it is important to highlight that these studies enrolled elderly cancer patients who may have already been experiencing age-related muscle loss, potentially introducing bias when evaluating the skeletal muscle outcomes related to cancer and chemotherapy [43,98,99].

Aerobic exercise induces significant metabolic remodeling in skeletal muscle, primarily characterized by the upregulation of the transcriptional cofactor PGC-1α, which plays a crucial role in mitochondrial biogenesis. PGC-1α promotes an oxidative phenotype and, consequently, a shift of fast-contracting fibers into slow-contracting ones. This metabolic energy sensor in skeletal muscle [100] protects against atrophy induced by age, disuse, and inflammation [101]. Other regulatory roles have been attributed to PGC-1α, such as the inhibition of FOXO3 and NF-kB signaling, providing a potential protective mechanism against muscle loss [31,34,100]. By inhibiting these pathways, voluntary physical activity in a running wheel was shown to promote the downregulation of atrogin-1 and MuRF1, E3 ligases from the UPS, in muscle tissue from mice [97], and 21–28 days of endurance exercise training (treadmill 5 days/week, 40–60 min, at 60% maximum speed) promoted the downregulation of NF-kB signaling in rats undergoing treatment with doxorubicin [32]. Aerobic exercise has also been associated with the upregulation of the insulin-like growth factor 1 (IGF-1)/PI3K/AKT/mTOR pathway [32,77], which was reported to be downregulated in the BC setting [58]. This pathway stimulates protein synthesis and muscle growth while simultaneously inhibiting FOXO3 transcriptional activity, thus decreasing the expression of E3 ligases [102] (Figure 2). Moreover, in rats inoculated with Walker-256 tumor cells, 52 days of resistance exercise (4–8 ladder climbs with 50%; 75%; 90%, and 100% maximal carrying capacity, 3 days/week) [103] was able to trigger protein synthesis, counteracting the increased proteolysis underlying cancer-induced muscle wasting [104]. This increase in protein synthesis may be mediated by mTOR pathway, which was observed to be upregulated following resistant exercise [103]. Notably, in one of the few molecular studies performed with BC patients undergoing chemotherapy and submitted to a combined exercise protocol (3 days/week, 90 min/day, 6 resistance exe plus ergometer bicycle), a significant increase in FOXO3, atrogin-1, and MuRF-1 expression was reported in *vastus lateralis* [29]. Nevertheless, immediately following resistance exercise, protein synthesis may be temporarily suppressed, which rapidly shifts moments after exercise [105]. These findings were reported in healthy fasted individuals, suggesting that the shift to anabolic metabolism was only promoted by exercise and not by feeding [105]. Even though an increase in IGF-1 signaling was shown after resistance exercise, there was no rise in the serum levels of this growth factor. Thus, it was proposed that the increased mechanical load induced by resistance exercise may activate IGF-1 signaling in skeletal muscle, which, in turn, induces the proliferation and differentiation of satellite cells, contributing to their activation and myonuclear accretion, as well as muscle mass gain [106] (Figure 2).

The changes in the levels of sex hormones accompanying BC can also impact muscle mass and function. Testosterone is known to directly activate mTOR pathway, resulting in increased protein synthesis and, eventually, muscle hypertrophy, in both type I and II muscle fibers [107]. Notably, some of the testosterone roles in male patients were not consistently reported in female ones, as the latter typically rely on estrogens to maintain skeletal muscle function [108]. Interestingly, estrogen seems to regulate fatigue and muscle contractile response in patients of both sexes. In fact, due to menopause, women often undergo a more pronounced and early decline in muscle strength compared to men [109]. However, in postmenopausal woman, progesterone may act similarly to testosterone, modulating muscle protein synthesis [110]. Moreover, estrogen may be implicated in enhancing skeletal muscle mass by promoting satellite cells activation and myonuclear accretion [111]. In BC patients, menopause is primarily associated with low estrogen levels, and these reduced levels are not as strongly associated with the adaptive changes promoted in muscle by resistance training compared to testosterone [112]. Different adaptations to resistance exercise were observed in healthy young women during different phases of the menstrual cycle, reporting different outcomes on muscle strength and power. In fact, these results showed that comparing luteal and follicular phase, the latter showed higher muscle gain and hypertrophy [113]. Similarly, in women performing any form of hormonal birth control, there were also reported diverse skeletal muscle outcomes [114]. Hence, this correlation underscores the importance of sex hormones fluctuations in resistance training induced adaptations and performance. Estrogen seems to improve overall muscular strength, whereas progesterone seems to negatively affect the muscle function in premenopausal women. Furthermore, it is important to acknowledge that in BC patients undergoing menopause, the low estrogen levels may lead to more subtle skeletal muscle outcomes and adaptations to resistance exercise training. However, and despite those changes in exercise adaptation, resistance training seemed to consistently increase overall muscle mass and strength [20].

## 4. Conclusions and Future Perspectives

Our review brings a novel perspective by focusing on muscle remodeling influenced by the interplay between BC, chemotherapy, and exercise, while examining the impact of different exercise programs. A spectrum of molecular mechanisms is responsible for muscle wasting in both cancer and chemotherapy, which can be mitigated to some extent by exercise training, thereby supporting its prescription. In BC, chemotherapy appears to exert a more pronounced impact on skeletal muscle remodeling than the cancer itself, leading to poorer health outcomes. Notably, inflammation is a common trigger for muscle wasting in both cancer and chemotherapy. Thus, the pathways modulated by pro-inflammatory cytokines play a key role in the imbalance between protein synthesis and breakdown, as well as in satellite cells’ activation and myonuclear accretion. Despite its recognized anti-inflammatory effects, the health benefits of aerobic exercise are equaled or even exceeded by resistance exercise in terms of increasing mass and strength and improving quality of life. Indeed, most studies that have investigated the therapeutic value of exercise training in BC patients undergoing chemotherapy have focused primarily on quality of life and functional parameters, with only a limited number of studies including molecular analyzes of skeletal muscle biopsies. Some investigations have been conducted in animal models; however, the interaction between chemotherapy and cancer in skeletal muscle remodeling remains understudied within the context of BC. In fact, the primary limitation of our study is the scarcity of research addressing the impact of exercise on BC and chemotherapy-induced muscle wasting. Therefore, to develop effective exercise regimens for BC patients at different stages, additional preclinical and clinical studies focused on muscle remodeling are essential.

Despite the recognized importance of skeletal muscle wasting in cancer management, it has received less attention compared to other organs such as the heart in response to cancer and chemotherapy. Therefore, it is crucial to undertake additional molecular research using animal models of BC subjected to various chemotherapy regimens. This research will help bridge existing knowledge gaps and provide a deeper insight into the skeletal muscle remodeling processes influenced by the interplay between BC and chemotherapy.

Moreover, future controlled clinical trials need to be conducted, enrolling BC patients at various disease stages and undergoing diverse exercise programs tailored to the individual condition of each patient. These trials should evaluate muscle wasting alongside cancer comorbidities to better understand muscle remodeling and maximize the benefits of exercise in managing BC patient’s health. This presents a significant challenge, given the diversity of cancer patients and the large cohorts required, as some BC patients may be unable or unwilling to participate in exercise programs. However, these clinical trials are crucial to produce guidelines that delineate personalized exercise regimens tailored to the individual needs of each BC patient. Furthermore, addressing barriers to implementing exercise in clinical practice, such as healthcare provider education, better patient motivation, and patients’ accessibility to structured exercise programs, should be the next step. Exercise has the potential to better manage cancer comorbidities and significantly improve the quality of life for BC patients. By reversing muscle mass loss and improving muscle quality, we anticipate better disease and treatment outcomes, considering the critical role of skeletal muscle in regulating overall health.

## Figures and Tables

**Table 1:** Overview of exercise protocols employed in preclinical models of cancer and/or chemotherapy and in clinical studies involving BC patients and reported outcomes.

Article	Randomization	Exercise	Muscle	Cancer Stage	Chemotherapy	Data Collected	Outcomes
**Pre-Clinical Studies**							
Padrão et al. [31]	Female Sprague-Dawley rats injected with N-Methyl-N-nitrosourea (MNU; 50 mg/kg) at 50 days of age and divided into 2 groups: MNU + SED; MNU + EX	Started 2 days after tumor induction and performed for 35 weeks on a treadmill (20 m/min; 60 min/day; 5 days/week).	*Gastrocnemius*	Mammary lesions (induced by MNU (50 mg/kg)with 100% incidence	No treatment	BW and muscle mass morphometry (CSA) serum inflammation and metabolic markers muscle inflammation and metabolic remodeling	↑ CSA after EXER; ↓ serum TWEAK after EXER; ↑ muscle ATP synthase beta/GAPDH, PGC1α, p-Akt/total Akt, 4E-BP1 after EXER; ↓ muscle NF-kB signaling
Alves de Lima et al. [32]	C57 BL/6 mice were subcutaneously implanted with Lewis lung carcinoma cells (8–10 wks old) divided into 3 groups: LLC; LLC + DOX; LLC + DOX + EXER	Started 1 wk after tumor inoculation and performed for 2 wk (21 d) or 3 wk (28 d). Aerobic: 5 times/wk, 40–60 min, on treadmill at 60% oxygen consumption	*Gastrocnemius*	Syngeneic model but not with BC	Doxorubicin 2 times/wk (cumulative dose 10 mg/kg)	BW and composition; Serum parameters (glycemia; lactate; TG; total cholesterol); glucose and insulin test; cytokine expression; biochemical markers of metabolic pathways.	↑ Muscle mass after EXER; EXER: = Serum parameters; EXER: = glucose tolerance and insulin sensitivity; EXER (28 d): = Fbxo32, Trim63 and myostatin expression; EXER (21 d): ↑ FoxO1; EXER (28 d): = FoxO1 and FoxO3; EXER (21 d): ↓ NFkB and ↓ proteolytic activity; EXER: = TLR-4.
Ballarò et al. [33]	Female and male BALB/c mice (6 wks old) divided in 4 groups: Control (n = 7), C26 (n = 6), C26 OXFU (n = 8) and C26 OXFU ex (n = 8) C26 mice were subcutaneously implanted with Colon 26 carcinoma cells	Combined: 5 times/wk for 28 d in custom motorized wheel. Started 5 d before tumor injection at 5 m/min for 15 min, and ↑ until 11 m/min for 45 min.	*Gastrocnemius*; *Tibialis anterior*	Syngeneic model but not with BC	Oxaliplatin (6 mg/kg) followed (2 h later) by 5-fluorouracil (50 mg/kg), weekly (for 28 d) starting 7 d after tumor injection.	BW; flow cytometry; hematocrit; MS. Muscle protein synthesis (surface sensing of translation method); biochemical assay.	C26 ex and C26 OXFU ex: counteract MW, by ↑ MS and mass; =hematocrit between groups; =IL-6 between groups; C26 OXFU ex: =STAT3, ↓ Fbxo32 and Trim63 (*Gastrocnemius*); C26 OXFU ex: ↓ Beclin-1, LC3B and p62 (*Gastrocnemius*); C26 OXFU ex: ↑ PGC-1α, Cyt-c and SDH (*Gastrocnemius*); C26 OXFU ex: ↑ Mfn2, whereas =Fis1 and Mfn1 (*Gastrocnemius*).
Smuder et al. [34]	23 male adult (6 month old) Sprague-Dawley rats, divided by 4 groups SED (n = 7); EXETR (n = 6); SEDDOX (n = 5); EXDOX (n = 6)	Aerobic: Treadmill running 5 times/wk (10, 20, 30, 40, 50 min/d on days 1–5). After 2 d rest, performed 60 min/day at 30 m/min (70% oxygen consumption) for more 5 d.	*Soleus*	No cancer	Doxorubicin (20 mg/kg), administrated 24 h before sacrifice	Histological analysis (H&E, apoptosis); biochemical assay.	EXEDOX: ↓ muscle fiber damage; EXEDOX: ↓ Beclin-1, LC3 and ATG12; EXEDOX: ↓ cathepsins.
Bredahl et al. [35]	60 Sprague-Dawley rats, were divided in 4 groups (RT (n = 20); TM (n = 20); SED (n = 20)). After the exercise protocol were divided in 6 groups of 10 rats (RT + SAL;TM + SAL;SED + SAL;RT + DOX; TM + DOX; SED + DOX)	A total of 10 wks. RT: Jump to reach an elevated food and water container, with ↑height during the wk until reaching 8 inches by wk 8. TM: Progressive treadmill training until 60 min, 30 m/min and 18°, representing 65–75% of oxygen consumption.	*Soleus*; *EDL*	No cancer	A single doxorubicin administration (15 mg/Kg) after the 10 wk exercise program	BW and composition; MF, MS (ex vivo skeletal muscle function).	TM + DOX: ↓ BW and ↓ *EDL* mass; RT + DOX: = BW and ↑ *EDL* mass; TM + DOX: = MS (*Soleus* and *EDL*); RT + DOX: ↑ MS (*Soleus*); RT + SAL and TM + SAL: ↓ MF (*Soleus*).
Quinn et al. [36]	47 male Sprague-Dawley rats (10 wk old) divided in 2 groups: SED (n = 20); EXER (n = 27), after 2 wks they were separated into 4 groups: SED + SAL (n = 10); SED + DOX (n = 10); EXER + SAL (n = 13); EXER + DOX (n = 14)	Aerobic: Progressive treadmill protocol, starting at 30 m/min for 10 min by wk 1, and ↑ until 60 min by wk 2.	*Soleus*; *EDL*; *DIA*	No cancer	A single doxorubicin administration (15 mg/Kg) 24 h after the last exercise session	BW and composition; Biochemical assays.	EXER groups: = *Soleus* mass; EXER + DOX: ↑ EDL mass than SED + DOX; EXER + DOX: ↑ Myf5 and MyoD (*Soleus* and *DIA*) and ↑ Myf4 (*Soleus*).
**Clinical Studies**							
Courneya et al. [27]	242 women (49 ± 30 years old), only 219 reported the studied outcomes with 80 patients in each group (AET; RET; UC)	Started 1 wk before chemo. and ended 3 wk after last administration (17 wk) AET: at 60% VO_2max_* (up 10% every 6 wk until 80%) during 15 min (up 5 min every 3 wk until 45 min) RET: 2 sets of 8–12 reps of 9 exe. (60 or 70% the maximal strength), if 12 reps were performed load ↑ 10%	Not available	Breast Cancer in stage I to IIIA	Taxanes or non-taxanes	BW and composition; QoL and fatigue; RDI; MS (by RM); VO2peak, mL/kg/min.	QoL = between exercise types; RET: ↑ RDI; AET: ↑ fitness healthy; RET: ↑ MS.
Klassen et al. [37]	281 women with (57.1 ± 6.1 years old), divided into 5 groups (No CT (n = 105); Started CT (n = 91); Post neo-adj. CT (n = 31); Post adj. CT (n = 28); Healthy (n = 26))	Resistance: A total of 12 wk, 2 times/wk. For 60 min, 8 exe. with 1–3 sets, with weight to 8–12 reps (60–80% RM*). 1 min of rest between sets and if 12 reps were performed, the weigh ↑ 5%.	Not available	Breast cancer in stage 0 to III after lumpectomy or mastectomy	Taxanes and/or Anthracyclines and/or Herceptin	QoL and fatigue; MF (muscular fatigue index*); MS (isokinetic and isometric tests).	Resistance exercise during CT: ↑ RDI; in post neo-adj. CT: ↑ MF.
Wengström et al. [30]	240 women (18–70 years old) divided into 3 groups (AET (n = 80); CT (n = 80); UC (n = 80))	60 min, 2 times/wk, 16 wk, with 5 min warm-up in aerobic machine at 10–12 RPE* and 10 min cool-down with scratching exercises. AET: 20 min at 13–15 RPE, followed by 3 × 3 min bouts at 16–18 RPE interspersed with ~1 min of passive or active recovery. CT: 8 exe., 2 sets of 10–12 reps at 70% RM and ↑ to 80% if 12 reps were performed, and 3 × 3 min bouts of AET.	*Vastus lateralis*	Breast cancer in stage I to IIIa undergoing chemotherapy	Taxanes or Anthracyclines	QoL and fatigue; MS (hydraulic hand dynamometer, isometric mid-thigh pull); secondary outcomes (contractile function; systemic inflammation; muscle fiber area; mitochondrial density; expression of genes for muscle adaptation).	AET ↓ cancer-related outcomes; in 1-year follow-up, an improvement in MS was still shown. In the 2-year follow-up, a significant maintaining of MS only in the CT group was shown; secondary outcomes not reported.
Schmidt et al. [28]	67 women (18–70 years old) divided into 3 groups (RT (n = 21); ET (n = 20); SC (n = 26))	2 times/wk, 60 min, 12 wk. ET: 10 min warm-up, followed by 25–30 min exercising on at 11–14 RPE, and 10 min cool-down. RT: 10 exe. at 1 RM to complete 20 reps.	Not available	Breast cancer with moderate- or high-risk	Adj. chemotherapy	QoL and fatigue; MS (isometric muscular capacity with M3 Diagnos); endurance stress test on muscles.	RT: ↑ RDI; RT: ↑ fatigue and QoL; RT: ↑ MS.
Møller et al. [29]	10 patients (28–62 years old). Biopsy only from 6 patients	Combined: 3 times/wk, 90-min, 10 wk. A session of 6 resistance exe. at 1 RM (progressively increasing), 10 min break, and patients consumed a whey protein drink (360 kcal), plus group sessions on ergometer bicycles (progressively increasing intensity).	*Vastus lateralis*	Breast cancer (n = 7), head and neck cancer (n= 1), rectal cancer (n = 1), sarcoma (n = 1)	Epirubicin and/or cyclophosphamide and/or doxorubicin/adramycin and/or carboplatin and/or vinorelbin/navelbine and/or flouroacil and/or trabectedin and/or gemcitabin	MS (performance evaluation); fiber CSA; biochemical markers of metabolic pathways; satellite cell analysis.	↓ type II fiber CSA during chemo., and ↑ with CT; ↑ GLUT4 during chemo. and after exercise; = AMPK and mitochondrial protein levels (Cyt-C, COX-IV, PDH, SDH, and VDAC); ↓ FOXO3 after CT; ↓ MuRF1 and atrogin-1 during chemo. and return to baseline after CT; ↑ mTOR during chemo and return to baseline after CT.
Winters-Stone et al. [38]	144 women ≥65 years old, divided into 3 groups: AET (n = 37), RET (n = 39) or FLEX (n = 38)	Supervised for 12 months, for 60-min, 3 times/wk: AET: low-impact dance, progressed from 20 to 45 min and increasing from 35% to 65% of HRR*. RET: 10 exercises, 2–3 sets of 10–15 reps at RM, (progressively increasing); FLEX: Stretching and relaxation exercise, 2–3 reps, holding for 15–60 s; Unsupervised for 6 months, with DVD-based version of their program, 3 times/wk.	Not available	Breast cancer stage I-III.	≥2 years postchemotherapy and/or radiation therapy	Physical function (SPPB*); physical fitness (6 MWD*); MS; flexibility.	AET and RET: ↑ physical performance; AET and RET: ↑ MS; RET: ↑ 6 MWD and SPPB; RET and FLEX: ↑ flexibility; RET unsupervised: ↑ lower extremity function.

*VO_2max_, maximal oxygen consumption (indicator of cardiovascular fitness and aerobic endurance); *RM, repetition maximum (maximum load a muscle group can overcame for a given number of repetitions) [39]; *RPE, rating of perceived exertion (on Borg Scale (0–20), measure how hard the activity is physically); *HRR, heart rate reserve (difference between your maximum heart rate and resting heart rate); *Muscular fatigue index: FI% = [(peak torque of initial three repetitions peak torque of final three repetitions)/peak torque of initial three repetitions] × 100, an adapted formula, described by Kannus [40]; *SPPB, Physical Performance Battery (3 timed performance tests scored 0–4. The scores are summed, where scores indicate better physical function and low scores on the SPPB (≤9) predict disability, hospitalization, nursing home admission, and mortality); *6MWD, 6 min walked distance (indicates mortality and morbidity in older adults and clinical populations); Abbreviations: 4E-BP1, Eukaryotic translation initiation factor 4E; ACC, Acetyl-CoA carboxylase; AET, aerobic exercise training; AMPK, AMP-activated protein kinase; ATG, Autophagy related; BW, Bodyweight; COX-IV, Cytochrome C Oxidase Subunit 4; CSA, cross-sectional area; CT, Combined training; Cyt-c, cytochrome c; DIA, diaphragm muscle; EDL, extensor digitorum longus; ET, endurance training; EXER, exercise group; Fbxo32/MAFbx, F-box only protein 32; Fis1, Fission 1; FLEX, flexibility; GAPDH, Glyceraldehyde 3-phosphate dehydrogenase; GLUT4, glucose transporter 4; HB, Home-based; IL-6, Interleukin-6; LC3B, Microtubule-associated protein 1A/1B-light chain 3; Mfn1/2, Mitofusin-1/2; MF, muscle fatigue; MNU, N-Nitroso-N-methylurea; MS, Muscle strength; mTOR, mammalian target of rapamycin; MuRF1, Muscle-specific RING finger protein 1; Myf, Myogenic factor; MyoD, Myogenic differentiation; NF-kB, Nuclear factor kappa-light-chain-enhancer of activated B cells; OXFU, Oxaliplatin; p62, Sequestosome-1; PGC-1α, Peroxisome proliferator-activated receptor-gamma coactivator; PDH, Pyruvate dehydrogenase; QoL, Quality of life; RDI, Relative dose intensity (measure chemotherapy completion rate); RET, Resistance exercise training; RT, resistance training; SAL, saline; SDH, Succinate dehydrogenase; STAT3, Signal transducer and activator of transcription 3 S6rp, Ribosomal protein S6; TG, triglyceride; TLR-4, Toll-like receptor 4; TM, Treadmill trained; TWEAK, TNF-related weak inducer of apoptosis; UC, usual care; ULK1, Unc-51-like kinase 1; VDAC, voltage-dependent anion channel; VO2peak, peak volume of oxygen consumed; ↑ increased; ↓ decreased.

## Data Availability

Not applicable.
